# Custom Foot Orthoses: A Retrospective Analysis of 1000 Prescriptions From New Zealand Podiatrists

**DOI:** 10.1002/jfa2.70044

**Published:** 2025-04-03

**Authors:** Aaron Jackson, Kelly Sheerin, Duncan Reid, Tim Ganley, Ben Lamb, Matthew R. Carroll

**Affiliations:** ^1^ Faculty of Health & Environmental Sciences School of Clinical Sciences Auckland University of Technology Auckland New Zealand; ^2^ Sports Performance Research Institute New Zealand (SPRINZ) Auckland University of Technology Auckland New Zealand; ^3^ Active Living and Rehabilitation: Aotearoa New Zealand Health and Rehabilitation Research Institute School of Clinical Sciences Auckland University of Technology Auckland New Zealand; ^4^ GO Orthotics Limited North Shore Mail Centre Auckland New Zealand; ^5^ FootMed Orthotic Lab Christchurch New Zealand

**Keywords:** custom foot orthoses, foot, orthoses prescription, plantar heel pain, podiatry

## Abstract

**Background:**

Podiatrists frequently prescribe foot orthoses to manage a range of musculoskeletal complaints. This study aimed to understand what characteristics were included in the design of custom foot orthoses, how symmetrically these were applied and whether there was an association between these features and the clinical diagnosis.

**Methods:**

One thousand orthotic prescriptions were obtained from two commercial orthotic labs in New Zealand. Twenty‐six prescription characteristics were analysed. Descriptive data detailed the frequency of included characteristics. The symmetry feature was derived according to the characteristics of both feet and analysed considering the number of times the left foot differed from the right foot. Clinical diagnoses were grouped, and for the most common four (plantar heel pain, pes planus, posterior tibial tendon and ankle sprain), associations between the prescription characteristics selected and diagnosis were analysed using cross tabulations and chi‐squared tests.

**Results:**

The most common prescription characteristics were a Polyamide 11 shell (80%), a shell thickness of 3 mm (54%), modified root shell style (61%) and varus cast correction (64%). Additionally, deep heel cups (36%), medial rearfoot (Kirby) skives (36%) and lateral forefoot wedges (22%) were the most prescribed modifications. Fifty‐eight percent of prescriptions were identical between the left and right sides. The most common diagnosis was plantar heel pain (11%). Plantar heel pain was associated with the characteristics of plantar fascia groove (*p* < 0.001), forefoot lateral wedge (*p* < 0.001) and heel cushion (*p* < 0.001).

**Conclusion:**

Strong associations between orthotic design characteristics and diagnoses indicate consistency in prescription variables amongst New Zealand podiatrists when prescribing custom foot orthoses. Plantar heel pain and pes planus are the two clinical diagnoses for which podiatrists prescribe the most custom foot orthoses. The high degree of similarity and symmetry in the prescription of orthoses raises questions regarding the specificity of prescriptions and opens a potential for future research on the topic.

**Abbreviations**
CFO: custom foot orthotic/orthosis
EVA: ethylene vinyl acetate
NZ: Aotearoa/New Zealand
PA11: Polyamide 11
PFO: prefabricated foot orthotic/orthosis
PHP: plantar heel pain
PMP: plantar metatarsal pad

## 1 Background

Foot orthoses are frequently prescribed for the management of various lower limb pathologies. The term ‘orthotic’ relates to any extrinsic device applied to the body to support, assist or improve movement [1]. Foot orthoses specifically relate to insoles placed in a person's shoe. These can be broadly categorised into two groups: custom foot orthoses (CFO) and prefabricated foot orthoses (PFO) [2]. Custom foot orthoses are differentiated from PFOs in that they are most often created from an impression, cast or scan of a patient's foot. Generally, these CFOs are constructed by a commercial orthotics laboratory following an orthotic prescription typically from a podiatrist.

Clinically, a distinguishing feature of CFOs, in contrast with PFOs, is the degree of specificity available in the construction process [3]. Clinicians can utilise an extensive range of modifications and design features according to what they deem most beneficial for their patients. Research regarding the influence of foot orthoses has explored the impact of individual orthotic characteristics on a vast range of biomechanical outcomes, such as kinematics and kinetics of the foot and lower limb, plantar pressure distribution and foot centre of pressure movement [4, 5, 6, 7, 8, 9]. However, only a small number of studies have investigated how clinicians develop their overall orthotic design, and none of these have been in the context of Aotearoa/New Zealand (NZ) [3, 10, 11].

Previous data have shown that NZ podiatrists prescribe significantly fewer CFOs than PFOs at a rate of nearly three to one [12, 13]. Chapman et al. [12] reported that NZ podiatrists prescribe 2.8 CFOs per week, unlike their Australian counterparts, who prescribe an average of 4.4 CFOs per week. In addition, several studies have examined these prescription habits in more detail [3, 10, 12, 14]. Menz et al. [3] analysed prescription variables submitted to an Australian CFO lab, categorising CFO prescriptions into three clusters. The authors identified significant differences between clusters concerning design features (orthosis type, cast correction, arch fill and rearfoot skives). Correlations were identified between the clusters and factors such as practice location, patient gender and age. Jackson et al. [14] surveyed podiatrists in NZ regarding their orthotic prescription habits, finding that respondents predominantly aimed to reduce tissue stress and frequently prescribed lateral forefoot wedges or metatarsal domes. Podiatrists responding to this survey suggested that root theory [15, 16] was the least influential podiatric paradigm when prescribing foot orthoses. However, many also indicated that they primarily utilise lateral wedging to ‘balance the foot’ [14].

This study aimed to investigate the CFO prescription habits of NZ podiatrists. Specifically, the study examined the following questions. First, what characteristics are frequently included in the orthotic prescription? Second, what is the left–right symmetry of orthoses within a prescription and which modifications are most commonly applied symmetrically? Finally, what is the relationship between the clinical diagnosis and characteristics included in the orthotic prescription?

## 2 Methods

### 2.1 Selection of Custom Foot Orthosis Prescriptions

One thousand orthotic prescriptions were sourced from two commercial orthotic labs (GO Orthotics Ltd. and FootMed Ltd.) based in NZ (500 from each lab). Each prescription represented one patient with possible multiple prescriptions in a small number of patients. Both labs supplied 500 consecutive prescriptions from their databases, working backwards from December 2021. The prescriptions supplied for analysis were submitted to the labs between August and December 2021 by approximately 70 different podiatrists. All data supplied were anonymised, and all patient and podiatrist identifiers were removed. Labs provided data using Microsoft Excel (Microsoft Corp, Redmond, Washington) on a templated spreadsheet developed by the first author (A.J.).

### 2.2 Custom Foot Orthosis Characteristics

**FIGURE 1:**
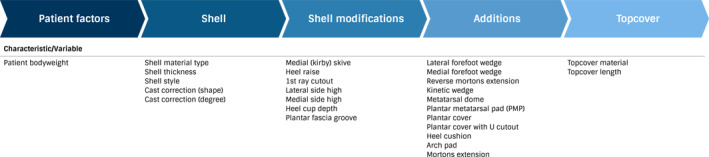
Prescription characteristics extracted from data.

Figure 1 displays the 26 prescription characteristics extracted from orthotic prescriptions for analysis. Data from the 26 prescription characteristics were pooled and analysed as a number (*n*) and percentage (%) out of 1000 prescriptions. Characteristics were recorded as included if they were present in the prescription for at least one foot (left, right or both).

### 2.3 Symmetry of Orthosis Prescriptions

The symmetry of prescriptions was analysed through two processes. First, the frequency of each orthotic characteristic being prescribed bilaterally (for both left and right sides) was compared to unilateral prescriptions (for only one side). Second, each prescription was evaluated as a whole to identify any differences between the left and right orthoses across all characteristics. These differences were then quantified, expressing the number and percentage of devices exhibiting varying degrees of asymmetry, from complete symmetry (no differences) to the highest degree of asymmetry observed in the dataset.

### 2.4 Grouping and Analysis of Diagnoses

Clinical diagnoses were classified into four categories: (i) plantar heel pain (PHP), (ii) pes planus, (iii) posterior tibial tendon pain/pathology and (iv) ankle sprain. Any diagnosis that referred to plantar fasciitis (including synonymous terms) or heel pain was classified as PHP. Pes planus included references to ‘planus feet’ or ‘flatfeet’. The posterior tibial tendon grouping included any diagnosis describing posterior tibial tendon pain, insufficiency or dysfunction. Finally, if the diagnosis referred to lateral ankle ligaments or lateral ankle instability, it was grouped under the ankle sprain diagnosis.

### 2.5 Data Analysis

Frequencies and proportions were used to describe the selected characteristics and diagnoses. An exploratory mixed effect model (SAS 9.4 proc glimmix procedure) was employed to analyse the most frequent diagnosis of PHP. This model accounted for potential correlations between the left and right foot by including each characteristic and participant as a random effect. The analysis revealed a high prevalence of symmetrical features among participants. Based on this finding, a symmetrical feature was derived and incorporated into the subsequent association analysis.

Associations between the four most common clinical diagnoses and CFO characteristics were assessed through cross‐tabulations and analysed in SPSS Statistics (version 29, IBM Corp., USA). The associations were evaluated using a chi‐squared test. To account for the large number of tests (and elevated risk of Type 1 and Type 2 errors), the significance threshold was adjusted using the Holm–Bonferroni method [17].

## 3 Results

### 3.1 Prescription Characteristics

Table 1 displays the frequency of prescription characteristics. The most commonly prescribed shell material was Polyamide 11 (PA11) (*n* = 801, 80%) and 3 mm was the most frequently selected shell thickness (*n* = 542, 54%). Most prescriptions included a modified root shell style (*n* = 614, 61%). The most common shell modifications included a deep heel cup (36%) (defined here as greater than 15 mm), ‘lateral side high’ (20%) and ‘medial side high’ (17%). Sixty‐four percent of prescriptions used a varus cast correction; approximately one‐third of prescriptions (*n* = 356, 36%) included a medial rearfoot (Kirby) skive, the most common skive depth being 4 mm (*n* = 192, 19%). A Spenco/Neolon top cover was the most frequently prescribed top cover material (*n* = 716, 72%), with 87% (*n* = 870) of orthoses designed with full‐length top covers.

**TABLE 1:** Frequency and symmetry of prescription characteristic**s**.

Characteristic	Included, *N* (%)	Symmetrical, *N* (%)
Shell style		
Modified root	614 (61)	^a^
Sagittal plane style	134 (13)	^a^
Medial STJ axis control (GO Orthotics only)	109 (11)	^a^
Other	142 (14)	^a^
Cast correction		
Varus	640 (64)	551 (86)
Valgus	56 (1)	34 (61)
Neutral	286 (29)	203 (71)
Shell material		
PA11	801 (80)	^a^
Polypropylene	128 (13)	^a^
EVA	69 (7)	^a^
Other	2 (0)	^a^
Shell thickness (*n* = 918)		
2.5 mm	60 (7)	^a^
3.0 mm	542 (59)	^a^
3.5 mm	150 (16)	^a^
4.0 mm	85 (9)	^a^
Other (2.0 mm, 4.5 mm or 5.0 mm)	81 (9)	^a^
Additions		
Lateral forefoot wedge	218 (22)	175 (80)
Arch pad	122 (12)	94 (77)
Medial forefoot wedge	118 (12)	4 (3)
Heel cushion	116 (12)	105 (91)
Reverse Morton's extension	90 (9)	67 (74)
Metatarsal dome	67 (7)	48 (72)
Plantar cover	59 (6)	48 (81)
Plantar metatarsal pad (PMP)	58 (6)	52 (90)
Modifications		
Lateral side high	196 (20)	149 (76)
1st ray cutout	176 (18)	141 (80)
Medial side high	167 (17)	146 (87)
Plantar fascia groove	63 (6)	52 (83)
Heel cup depth		
Deep (> 14 mm)	360 (36)	330 (92)
Shallow (< 11 mm)	68 (7)	63 (93)
Kirby skive		
All depths	356 (36)	257 (72)
2 mm	169 (17)	102 (60)
4 mm	192 (19)	137 (71)
> 4 mm	21 (2)	18 (86)
Topcover material		
Neolon/Spenco	716 (72)	^a^
Lunasoft	77 (8)	^a^
Leather	70 (7)	^a^
Other	129 (13)	^a^
Topcover length		
Full length	870 (87)	^a^
Sulcus (3/4)	80 (8)	^a^
Shell only	38 (4)	^a^

^a^ 100% symmetry between left and right orthoses.

### 3.2 Symmetry of Custom Foot Orthosis Prescriptions

Table 1 displays the prescription symmetry characteristics. The most symmetrically applied addition was heel cushions (poron applied on top of the shell at the heel), which, when included on one side, were also applied to the contralateral side in 91% of cases. Most modifications were highly symmetrical, with one distinct exception being medial forefoot wedges, applied unilaterally in 97% of the prescriptions in which they appeared. Table 2 presents an analysis of the characteristic of symmetry. Fifty‐eight percent (*n* = 575) of prescriptions contained no difference between the left and right sides. By comparison, 31% (*n* = 313) of prescriptions requested one or two differences between the left and right sides and the remaining 11% (*n* = 112) of prescriptions included between 3 and 7 asymmetries.

**TABLE 2:** Prescription symmetry.

Prescription differences between left and right orthoses	Prescriptions, *N* (%)
No differences	575 (58)
1 difference	202 (20)
2 differences	111 (11)
3 differences	54 (5)
4 differences	31 (3)
5 differences	15 (2)
6 differences	10 (1)
7 differences	2 (0)

### 3.3 Relationship Between the Clinical Diagnosis and Orthosis Characteristics

Most prescriptions (*n* = 937, 94%) included a clinical diagnosis. A complete list of diagnostic groups and the clinical diagnoses included is available in Table S1. The four most common clinical diagnoses that CFOs were prescribed for were PHP (*n* = 110, 11%), pes planus (*n* = 81, 8%), posterior tibial tendon pain or dysfunction (*n* = 61, 6%) and ankle sprains (*n* = 50, 5%). Table 3 presents the results of the significant associations between diagnosis and orthotic inclusions as part of the association analysis (Table S2). Table 3 presents only the significant associations where the prescription characteristic is more often included in the diagnosis. Those which were significant but less often included are not presented in this table. Each of the four most common diagnoses had several significant associations. Prescriptions related to posterior tibial tendon pain were less often (34%) symmetrical. No significant association was observed regarding symmetry for the other three common diagnoses.

**TABLE 3:** Significant comparisons between clinical diagnosis and prescription characteristics.

Diagnosis	Characteristic	Qualifier	Distribution when the diagnosis is selected (%)	Distribution when the diagnosis is not selected (%)	Peason chi‐squared	*p* value	Holm–Bonferroni corrected threshold	Significance after multiple tests correction
Plantar heel pain	Shell style	Mod root	74 (67.3)	54 (60.7)	14.701	0.002	0.002	Yes
		Sagittal plane style	23 (20.9)	111 (12.5)				
	Varus cast correction	No	29 (26.4)	331 (37.2)	4.981	0.026	0.002	No
		Yes	81 (73.6)	559 (62.8)				
	Plantar fascia groove	No	87 (79.1)	850 (95.5)	44.686	< 0.001	0.002	Yes
		Yes	23 (20.9)	40 (4.5)				
	Forefoot lateral wedge	No	72 (65.5)	710 (79.8)	11.777	< 0.001	0.002	Yes
		Yes	38 (34.5)	180 (20.2)				
	Heel cushion	No	65 (59.1)	819 (92)	103.537	< 0.001	0.002	Yes
		Yes	45 (40.9)	71 (8)				
Pes planus	Shell style	Medial STJ axis	27 (33.3)	82 (8.9)	47.183	< 0.001	0.002	Yes
	Kirby skive	No	44 (54.3)	600 (65.3)	3.905	0.048	0.003	No
		Yes	37 (45.7)	319 (34.7)				
	Kinetic wedge	No	78 (96.3)	909 (98.9)	3.969	0.046	0.003	No
		Yes	3 (3.7)	10 (1.1)				
	Arch pad	No	55 (67.9)	823 (89.6)	32.581	< 0.001	0.002	Yes
		Yes	26 (32.1)	96 (10.4)				
	Medial forefoot wedge	No	63 (77.8)	819 (89.1)	9.199	0.002	0.002	Yes
		Yes	18 (22.2)	100 (10.9)				
	Medial side high	No	57 (70.4)	776 (84.4)	10.592	0.001	0.002	Yes
		Yes	24 (29.6)	143 (15.6)				
	Deep heel cup	No	40 (49.4)	600 (65.3)	8.174	0.004	0.003	No
		Yes	41 (50.6)	319 (34.7)				
Posterior tibial	Shell style	Sagittal plane style	10 (16.4)	124 (13.2)	51.127	< 0.001	0.002	Yes
		Medial STJ axis	23 (37.7)	86 (9.2)				
	Varus cast correction	No	8 (13.1)	352 (37.5)	14.767	< 0.001	0.002	Yes
		Yes	53 (86.9)	587 (62.5)				
	Arch pad	No	42 (68.9)	836 (89)	21.773	< 0.001	0.002	Yes
		Yes	19 (31.1)	103 (11)				
	Kirby skive	No	21 (34.4)	623 (66.3)	25.457	< 0.001	0.002	Yes
		Yes	40 (65.6)	316 (33.7)				
	Medial forefoot wedge	No	45 (73.8)	837 (89.1)	12.996	< 0.001	0.002	Yes
		Yes	16 (26.2)	102 (10.9)				
Ankle sprain	Valgus cast correction	No	38 (76)	906 (95.4)	33.707	< 0.001	0.002	Yes
		Yes	12 (24)	44 (4.6)				
	Forefoot lateral wedge	No	26 (52)	756 (79.6)	21.193	< 0.001	0.002	Yes
		Yes	24 (48)	194 (20.4)				
	Lateral side high	No	20 (40)	784 (82.5)	54.513	< 0.001	0.002	Yes
		Yes	30 (60)	166 (17.5)				

*Note:* If a characteristic was included in the prescription on AT LEAST one foot, it is recorded here as ‘yes’. If the characteristic was not applied to either left or right foot, it was recorded as ‘no’.

## 4 Discussion

This study explored the CFO prescription habits of NZ podiatrists. Custom foot orthoses were frequently prescribed using a ‘Modified Root’ shell style, 3 mm PA11 (a polyamide bioplastic produced from caster beans and frequently used in additive manufacturing) [18], and a varus cast correction. Lateral forefoot wedges were the most commonly prescribed addition, and full‐length top covers were prescribed for most orthoses. Approximately 60% of orthoses had no difference in design characteristics between the left and right sides. The diagnostic groups PHP, pes planus, posterior tibial tendon pain and ankle sprains demonstrated strong statistical associations with orthotic characteristics, indicating some consistency between podiatrists when prescribing orthoses for these conditions.

The finding that lateral foot wedges are the most prescribed addition supports previous NZ‐based foot orthoses research conducted by Jackson et al. [14]. Data indicated that the most common shell style selection was the ‘Modified Root’, which is derived from the work of Merton Root and colleagues, now known as ‘Root Theory’ [15, 16, 19]. This finding conflicts with previous NZ research, indicating that the root theory was less likely to influence orthotic design decisions than the sagittal plane facilitation theory [14, 20]. First, this may be explained by this shell style's longstanding and accepted use [13, 21]. Secondly, prescription habits are based on podiatrists' beliefs stemming from their education and clinical experiences, and thirdly, the options provided by the labs on their prescription forms. This phenomenon is unlikely to be exclusive to the labs included in this study, as each lab that offers CFO fabrication requires the submission of a prescription form.

Similar to previous Australian research, the current study found a high level of symmetry among CFO prescriptions [3]. Almost 60% of the CFO prescriptions in the current study were designed identically between the left and right feet. Several previous studies, including Mills et al. [22] and Mundermann et al. [23], have identified a link between the orthotic design and perceived comfort. Thus, we postulate that the high degree of prescription symmetry may be an attempt to optimise comfort for the wearer if it is speculated that highly asymmetrical CFO designs with multiple differences on one limb compared to the other may be uncomfortable. Comfort is known to be both protective and improves tolerance and adherence to foot orthoses [24, 25]. Although individual preferences exist, arch height, heel cup fit and cushioning have been shown to improve comfort [25]. The two characteristics that most often applied symmetrically in the current study were a ‘heel cushion’ and a ‘plantar metatarsal pad’, features frequently considered to improve cushioning and comfort.

The high degree of prescription symmetry raises questions about the specificity of customised devices. With the ability to alter components of a CFO, why are most prescriptions symmetrical and only a small percentage containing one or two differences between body sides? When considering the concept of limb dominance and that humans often do not function in a biomechanically symmetrical way [26], we must consider why we do not see a greater degree of prescription variation in a device designed to be individually customised. The current results may indicate that highly symmetrical CFO prescriptions mean lower limb foot function is not being optimised or that podiatrists rely on the bespoke plantar contour of these devices to elicit an effect. Further research is needed to explore why podiatrists often prescribe symmetrical CFO and to investigate the biomechanical and comfort effects of asymmetrical designs.

The most common clinical diagnosis associated with orthotic prescriptions in the present study was PHP. This finding was not surprising as PHP is reported to affect between 4% and 7% of the adult population and is the most common musculoskeletal condition seen by podiatrists [27, 28, 29, 30, 31, 32, 33]. Due to the relatively high prevalence of PHP, researchers have paid great attention to the influence of foot orthoses on this condition. Whittaker et al. [33] concluded that foot orthoses reduce patient symptoms in the medium term and should be included in the management of this condition when initial inexpensive options have failed [33, 34]. A recent best practice guide for PHP by Morrissey et al. [27] also included the provision of foot orthoses (either PFO or CFO). Notwithstanding this population's frequent use of foot orthoses, guidance on optimal prescription and design decisions is unavailable. Based on the current study data, plantar fascia groove, forefoot lateral wedge and heel cushioning are considered key design features when managing PHP through orthotic therapy.

Data also indicated that pes planus and posterior tibial tendon pathology commonly led to CFO prescription. Although pes planus has been the topic of recent controversy regarding its significance [35], this morphological term describes a foot which has reduced arch height, calcaneal eversion in stance and lateral deviation of the forefoot [36, 37]. Pes planus is often suggested to be a contributing factor to posterior tibial tendon pathology, or in fact, developed due to insufficiency of the posterior tibial tendon [38]. For this reason, it is not surprising that both diagnoses were associated with similar CFO design inclusions. Arch pad and medial forefoot wedge are consistent across both diagnoses. Interestingly, a varus cast correction and Kirby skive were associated with posterior tibial tendon pathology; however, this was not the case for pes planus despite the rearfoot position being previously linked to pes planus [11, 36, 39, 40, 41, 42]. A Delphi study by Banwell et al. [39], exploring the prescription of orthoses for pes planus, also indicated that the Kirby skive is an important consideration, postulating that if the intention of the orthotic is to gain increased control, then a Kirby skive should be included. Medial forefoot wedges have been shown to shift the centre of pressure laterally in individuals with pronated feet, which could be expected to reduce the workload of the posterior tibial tendon [43].

Data indicated that a diagnosis of ankle sprain was significantly associated with a valgus cast correction, forefoot lateral wedges and ‘lateral side high’ shell modification. These characteristics are all associated with minimising excessive inversion of the foot and reducing the workload of muscles in the lateral lower leg and ankle region. For patients with a recent ankle sprain or chronic ankle instability, the benefit of an orthotic has been suggested to lie in the impact it may have on neuromuscular function and postural control [44]. Lateral wedges encourage rearfoot eversion as well as increase the external eversion moment, both of which may be protective for lateral ankle instability and the risk of lateral ankle sprains [6].

The study findings need to be interpreted whilst considering several limitations. Firstly, the data relate to prescriptions from 2021. Given the rapid and continuous advances in orthotic design and fabrication, particularly additive manufacturing, some content may already be outdated. Of note, 13% of prescriptions analysed used a Polypropylene shell, and yet, as of 2024, both labs that supplied these data have discontinued Polypropylene to focus solely on PA11 and EVA. Secondly, we analysed the clinical diagnosis provided by the referring podiatrist, although it was not recorded if this pathology was unilateral (if so, which side) or bilateral. The diagnoses were also unconfirmed clinical diagnoses and we do not know the outcomes of the CFOs prescribed. An interesting extension of this work would be to undertake a specific analysis of the common diagnoses, including confirmation of diagnosis and an outcome of the CFO. Finally, diagnoses were grouped manually using descriptions provided on the prescription form. Future work may benefit from more explicit diagnostic categories, which could be added as dropdown boxes to the laboratory's prescription form.

## 5 Conclusion

An analysis of 1000 CFO prescriptions demonstrated NZ podiatrists frequently prescribe orthoses manufactured from 3 mm PA11 using a modified root shell style with varus cast correction in the rearfoot. Plantar heel pain and pes planus are the two clinical diagnoses for which podiatrists prescribe the greatest number of CFOs. Strong associations between orthotic design characteristics and diagnoses indicate consistency in prescription variables amongst podiatrists when managing these conditions. The high degree of prescription symmetry raises questions about the specificity of customised devices and opens an avenue for future research.

## Author Contributions

**Aaron Jackson:** conceptualisation, data curation, formal analysis, methodology, writing – original draft preparation, writing–reviewing and editing. **Kelly Sheerin:** conceptualisation, methodology, writing – reviewing and editing. **Duncan Reid:** conceptualisation, methodology, writing – reviewing and editing. **Tim Ganley:** resources, methodology. **Ben Lamb:** resources, methodology. **Matthew R. Carroll:** conceptualisation, formal analysis, methodology, writing – original draft preparation, writing – reviewing and editing.

## Ethics Statement

After completing an online ethics question matrix hosted by the AUT University Ethics Committee, it was determined that the ethics approval was not required for this study. Ethical approval was not sought due to the data being nonsensitive and anonymous. The researchers did not contact participants, and each lab involved in the study sent a notice to their clients with an opportunity to have their prescriptions excluded from the analysis. Nonresponse to this request was considered consent.

## Conflicts of Interest

M.C. is an Associate Editor for the Journal of Foot and Ankle Research. All other authors declare no conflicts of interest.

## Supporting information

Table S1

Table S2

## Data Availability

Requests for further details of the dataset and queries relating to data‐sharing arrangements may be submitted to Aaron Jackson (aaron.jackson@aut.ac.nz).
