# Supplementary material for: Custom Foot Orthoses: A Retrospective Analysis of 1000 Prescriptions From New Zealand Podiatrists
Source: J Foot Ankle Res. 2025 Apr 3;18(2):e70044. doi: 10.1002/jfa2.70044 (PMC11967362; doi:10.1002/jfa2.70044)
Supplement: Supplementary file 1 — Table S1 [file JFA2-18-e70044-s002.docx]

Appendix 1: Diagnostic groups and their associated definitions

| **Diagnosis** | **N (%)** | **Description** |
| --- | --- | --- |
| PHP | 110 (11) | Plantar heel pain including all references to the plantar fascia or "heel pain" |
| Pes Planus | 81 (8) | Pes Planus including pes planovalgus, flat foot and 'collapsed arches' |
| Posterior tibial tendon (Incl PTTD) | 61 (6) | Tibialis posterior tendon pain, tendinopathy, insufficiency, or PTTD (any stage) |
| Ankle sprain | 50 (5) | Lateral ankle sprain or lateral ankle instability |
| Achilles | 45 (5) | Achilles tear, strain, rupture, tendinopathy or tendinosis |
| Forefoot pain | 41 (4) | Forefoot pain including MPJ pain, forefoot discomfort, metatarsalgia, excluding bursitis, mortons neuroma, and 1st MPJ joint sprain |
| Knee injury/pain | 34 (3) | Knee injury or pain including osteoarthritis, PFPS, ITB, Meniscus injury |
| Replacement orthotics | 30 (3) | Replacement pairs of orthotics without mention of pathology or another clinical diagnosis |
| Ankle pain (Excluding ligament injury, sprain or ankle surgery/reconstruction) | 30 (3) | Ankle fracture or ankle pain with no reference to ligaments |
| HAV | 28 (3) | Hallux abducto valgus |
| Arch pain or medial midfoot pain | 28 (3) | Arch pain or generalised midfoot pain |
| Overpronation | 28 (3) | Pronation, overpronation, valgus rearfoot |
| Ankle surgery | 27 (3) | Orthoses prescribed post ankle reconstructions or stabilisation surgery |
| Hallux Limitus | 22 (2) | Structural hallux limitus/rigidus or functional hallux limitus/rigidus |
| Shin injury/pain | 20 (2) | Shin pain, shin splints, MTSS |
| Lisfranc | 19 (2) | Lisfranc fracture or injury |
| Mortons Neuroma | 19 (2) | Neuroma or mortons neuroma |
| Peroneal tendon issue | 17 (2) | Peroneal tendon injury, tendinopathy or strain |
| Pes Cavus | 17 (2) | Pes Cavus, High Arch, Cavovarus, or any description of a high arched foot |
| 1st MPJ pain/injury | 16 (2) | 1st MPJ joint sprain |
| Kinematic/Anatomical Dx | 15 (2) | Broad category including diagnoses of poor biomechanics with no reference to pain or symptoms |
| Foot injury (no more specific) | 15 (2) | Generalised foot pain or tender feet |
| Neuropathy or neurological conditions | 15 (2) | Neuropathy or neurological condition |
| Post surgical | 14 (1) | A broad range of post surgical cases including bunionectomy, reconstructions, skin grafts, and amputations |
| Diabetes | 14 (1) | Diabetes foot |
| Lateral midfoot pain | 13 (1) | Lateral foot pain including cuboid pain/syndrome |
| Other | 12 (1) | Non specific fractures, blistering |
| Rheumatoid Arthritis | 12 (1) | Rheumatoid arthritis |
| Instability | 7 (1) | Instability (excluding lateral ankle instability) |
| Back pain | 7 (1) | Back pain |
| Motor vehicle accident | 7 (1) | Motor vehicle accident |
| Hip injury/pain | 7 (1) | Hip pain or hip surgery |
| Bursitis | 7 (1) | Forefoot bursitis |
| Calcaneus fracture | 7 (1) | Calcaneal fracture |
| Rearfoot pain | 6 (1) | General rearfoot pain excluding calcaneal fractures and PHP or any reference to the plantar fascia |
| Sesamoiditis | 6 (1) | Sesamoiditis |
| Limb length discrepancy | 6 (1) | Limb length discrepancy |
| Sinus tarsi syndrome | 6 (1) | Sinus tarsi syndrome |
| Paediatric Flatfoot | 3 (0) | Paediatric flatfoot |
| Tibialis Anterior | 2 (0) | Tibialis anterior strain or injury |
| Clubfoot | 1 (0) | Clubfoot |
| Calf injury/pain | 1 (0) | Gastroc strain |
