# Supplementary material for: Custom Foot Orthoses: A Retrospective Analysis of 1000 Prescriptions From New Zealand Podiatrists
Source: J Foot Ankle Res. 2025 Apr 3;18(2):e70044. doi: 10.1002/jfa2.70044 (PMC11967362; doi:10.1002/jfa2.70044)
Supplement: Supplementary file 2 — Table S2 [file JFA2-18-e70044-s001.docx]

**Appendix 2:** Cross tabulations and association analysis of all orthotic characteristics in relation to each of the top four diagnoses

- Comparisons which meet the criteria for significance after Holm-Bonferroni correction are highlighted in green.
- Comparisons which have a P value < 0.05 but are not significant after Holm-Bonferroni correction are highlighted in yellow.

**Plantar heel pain**

| **Characteristic** | **Qualifier** | **PHP (%)** | **Not PHP (%)** | **Peason Chi-Square** | **P value** | **Holm-Bonferroni corrected threshold** | **Significance after multiple tests correction** |
| --- | --- | --- | --- | --- | --- | --- | --- |
| Shell thickness | Not reported | 7 (6.4) | 75 (8.4) | 6.693 | 0.462 | 0.005 | No |
|  | 2 mm | 2 (1.8) | 35 (3.9) |  |  |  |  |
|  | 2.5 mm | 7 (6.4) | 53 (6) |  |  |  |  |
|  | 3 mm | 58 (52.7) | 484 (54.4) |  |  |  |  |
|  | 3.5 mm | 24 (21.8) | 126 (14.2) |  |  |  |  |
|  | 4 mm | 7 (6.4) | 78 (8.8) |  |  |  |  |
|  | 4.5 mm | 5 (4.5) | 36 (4) |  |  |  |  |
|  | 5 mm | 0 (0) | 3 (0.3) |  |  |  |  |
| Shell style | Mod Root | 74 (67.3) | 54 (60.7) | 14.701 | 0.002 | 0.002 | Yes |
|  | Sagittal Plane | 23 (20.9) | 111 (12.5) |  |  |  |  |
|  | Medial STJ | 6 (5.5) | 103 (11.6) |  |  |  |  |
|  | Other | 7 (6.4) | 136 (15.3) |  |  |  |  |
| Varus cast correction | No | 29 (26.4) | 331 (37.2) | 4.981 | 0.026 | 0.002 | No |
|  | Yes | 81 (73.6) | 559 (62.8) |  |  |  |  |
| Valgus cast correction | No | 108 (98.2) | 836 (93.9) | 3.344 | 0.067 | 0.003 | No |
|  | Yes | 2 (1.8) | 54 (6.1) |  |  |  |  |
| Kirby Skive | No | 68 (61.8) | 576 (64.7) | 0.359 | 0.549 | 0.006 | No |
|  | Yes | 42 (38.2) | 314 (35.3) |  |  |  |  |
| Heel raise | No | 106 (96.4) | 828 (93) | 1.761 | 0.184 | 0.003 | No |
|  | Yes | 4 (3.6) | 62 (7) |  |  |  |  |
| 1^st^ Ray cutout | No | 84 (76.4) | 740 (83.1) | 3.105 | 0.078 | 0.003 | No |
|  | Yes | 26 (23.6) | 150 (16.9) |  |  |  |  |
| Lateral side high | No | 102 (92.7) | 702 (78.9) | 11.919 | < 0.001 | 0.002 | Yes |
|  | Yes | 8 (7.3) | 188 (21.1) |  |  |  |  |
| Medial side high | No | 86 (78.2) | 747 (83.9) | 2.327 | 0.127 | 0.003 | No |
|  | Yes | 24 (21.8) | 143 (16.1) |  |  |  |  |
| Deep heel cup | No | 66 (60) | 574 (64.5) | 0.858 | 0.354 | 0.004 | No |
|  | Yes | 44 (40) | 316 (35.5) |  |  |  |  |
| Shallow heel cup | No | 101 (91.8) | 831 (93.4) | 0.372 | 0.542 | 0.006 | No |
|  | Yes | 9 (8.2) | 59 (6.6) |  |  |  |  |
| Plantar fascia groove | No | 87 (79.1) | 850 (95.5) | 44.686 | < 0.001 | 0.002 | Yes |
|  | Yes | 23 (20.9) | 40 (4.5) |  |  |  |  |
| Forefoot lateral wedge | No | 72 (65.5) | 710 (79.8) | 11.777 | < 0.001 | 0.002 | Yes |
|  | Yes | 38 (34.5) | 180 (20.2) |  |  |  |  |
| Medial forefoot wedge | No | 93 (84.5) | 789 (88.7) | 1.586 | 0.208 | 0.003 | No |
|  | Yes | 17 (15.5) | 101 (11.3) |  |  |  |  |
| Reverse mortons extension | No | 98 (89.1) | 812 (91.2) | 0.550 | 0.458 | 0.005 | No |
|  | Yes | 12 (10.9) | 78 (8.8) |  |  |  |  |
| Kinetic wedge | No | 108 (98.2) | 879 (98.8) | 0.259 | 0.611 | 0.008 | No |
|  | Yes | 2 (1.8) | 11 (1.2) |  |  |  |  |
| Metatarsal dome | No | 106 (96.4) | 827 (92.9) | 1.856 | 0.173 | 0.003 | No |
|  | Yes | 4 (3.6) | 63 (7.1) |  |  |  |  |
| PMP | No | 105 (95.5) | 837 (94) | 0.356 | 0.551 | 0.007 | No |
|  | Yes | 5 (4.5) | 53 (6) |  |  |  |  |
| Plantar cover | No | 106 (96.4) | 835 (93.8) | 1.141 | 0.286 | 0.004 | No |
|  | Yes | 4 (3.6) | 55 (6.2) |  |  |  |  |
| Heel Cushion | No | 65 (59.1) | 819 (92) | 103.537 | < 0.001 | 0.002 | Yes |
|  | Yes | 45 (40.9) | 71 (8) |  |  |  |  |
| Arch pad | No | 98 (89.1) | 780 (87.6) | 0.192 | 0.661 | 0.010 | No |
|  | Yes | 12 (10.9) | 110 (12.4) |  |  |  |  |
| Mortons extension | No | 109 (99.1) | 869 (97.6) | 0.957 | 0.328 | 0.004 | No |
|  | Yes | 1 (0.9) | 21 (2.4) |  |  |  |  |
| Symmetrical | No | 46 (41.8) | 379 (42.6) | 0.024 | 0.878 | 0.013 | No |
|  | Yes | 64 (58.2) | 511 (57.4) |  |  |  |  |

**Pes Planus**

| **Characteristic** | **Qualifier** | **PP (%)** | **Not PP (%)** | **Peason Chi-Square** | **P value** | **Holm-Bonferroni corrected threshold** | **Significance after multiple tests correction** |
| --- | --- | --- | --- | --- | --- | --- | --- |
| Shell thickness | Not reported | 10 (12.3) | 72 (7.8) | 12.244 | 0.093 | 0.004 | No |
|  | 2 mm | 0 (0) | 37 (4) |  |  |  |  |
|  | 2.5 mm | 4 (4.9) | 56 (6.1) |  |  |  |  |
|  | 3 mm | 40 (49.4) | 502 (54.6) |  |  |  |  |
|  | 3.5 mm | 15 (18.5) | 135 (14.7) |  |  |  |  |
|  | 4 mm | 10 (12.3) | 75 (8.2) |  |  |  |  |
|  | 4.5 mm | 1 (1.2) | 40 (4.4) |  |  |  |  |
|  | 5 mm | 1 (1.2) | 2 (0.2) |  |  |  |  |
| Shell style | Mod Root | 40 (49.4) | 574 (62.5) | 47.183 | < 0.001 | 0.002 | Yes |
|  | Sagittal Plane | 9 (11.1) | 125 (13.6) |  |  |  |  |
|  | Medial STJ | 27 (33.3) | 82 (8.9) |  |  |  |  |
|  | Other | 5 (6.2) | 138 (15) |  |  |  |  |
| Varus cast correction | No | 24 (29.6) | 336 (36.6) | 1.552 | 0.213 | 0.004 | No |
|  | Yes | 57 (70.4) | 583 (63.4) |  |  |  |  |
| Valgus cast correction | No | 80 (98.8) | 864 (94) | 3.177 | 0.075 | 0.003 | No |
|  | Yes | 1 (1.2) | 55 (6) |  |  |  |  |
| Kirby Skive | No | 44 (54.3) | 600 (65.3) | 3.905 | 0.048 | 0.003 | No |
|  | Yes | 37 (45.7) | 319 (34.7) |  |  |  |  |
| Heel raise | No | 77 (95.1) | 857 (93.3) | 0.395 | 0.530 | 0.013 | No |
|  | Yes | 4 (4.9) | 62 (6.7) |  |  |  |  |
| 1^st^ Ray cutout | No | 70 (86.4) | 754 (82) | 0.982 | 0.322 | 0.006 | No |
|  | Yes | 11 (13.6) | 165 (18) |  |  |  |  |
| Lateral side high | No | 79 (97.5) | 725 (78.9) | 16.414 | < 0.001 | 0.002 | Yes |
|  | Yes | 2 (2.5) | 194 (21.1) |  |  |  |  |
| Medial side high | No | 57 (70.4) | 776 (84.4) | 10.592 | 0.001 | 0.002 | Yes |
|  | Yes | 24 (29.6) | 143 (15.6) |  |  |  |  |
| Deep heel cup | No | 40 (49.4) | 600 (65.3) | 8.174 | 0.004 | 0.003 | No |
|  | Yes | 41 (50.6) | 319 (34.7) |  |  |  |  |
| Shallow heel cup | No | 77 (95.1) | 855 (93) | 0.482 | 0.488 | 0.010 | No |
|  | Yes | 4 (4.9) | 64 (7) |  |  |  |  |
| Plantar fascia groove | No | 80 (98.8) | 857 (93.3) | 3.831 | 0.050 | 0.003 | No |
|  | Yes | 1 (1.2) | 62 (6.7) |  |  |  |  |
| Forefoot lateral wedge | No | 76 (93.8) | 706 (76.8) | 12.626 | < 0.001 | 0.002 | Yes |
|  | Yes | 5 (6.2) | 213 (23.2) |  |  |  |  |
| Medial forefoot wedge | No | 63 (77.8) | 819 (89.1) | 9.199 | 0.002 | 0.002 | Yes |
|  | Yes | 18 (22.2) | 100 (10.9) |  |  |  |  |
| Reverse mortons extension | No | 80 (98.8) | 830 (90.3) | 6.490 | 0.011 | 0.003 | No |
|  | Yes | 1 (1.2) | 89 (9.7) |  |  |  |  |
| Kinetic wedge | No | 78 (96.3) | 909 (98.9) | 3.969 | 0.046 | 0.003 | No |
|  | Yes | 3 (3.7) | 10 (1.1) |  |  |  |  |
| Metatarsal dome | No | 78 (96.3) | 855 (93) | 1.266 | 0.261 | 0.005 | No |
|  | Yes | 3 (3.7) | 64 (7) |  |  |  |  |
| PMP | No | 78 (96.3) | 864 (94) | 0.709 | 0.400 | 0.008 | No |
|  | Yes | 3 (3.7) | 55 (6) |  |  |  |  |
| Plantar cover | No | 78 (96.3) | 863 (93.9) | 0.766 | 0.382 | 0.007 | No |
|  | Yes | 3 (3.7) | 56 (6.1) |  |  |  |  |
| Heel Cushion | No | 75 (92.6) | 809 (88) | 1.511 | 0.219 | 0.005 | No |
|  | Yes | 6 (7.4) | 110 (12) |  |  |  |  |
| Arch pad | No | 55 (67.9) | 823 (89.6) | 32.581 | < 0.001 | 0.002 | Yes |
|  | Yes | 26 (32.1) | 96 (10.4) |  |  |  |  |
| Mortons extension | No | 78 (96.3) | 900 (97.9) | 0.926 | 0.336 | 0.006 | No |
|  | Yes | 3 (3.7) | 19 (2.1) |  |  |  |  |
| Symmetrical | No | 28 (34.6) | 397 (43.2) | 2.269 | 0.132 | 0.004 | No |
|  | Yes | 53 (65.4) | 522 (56.8) |  |  |  |  |

**Posterior tibial tendon**

| **Characteristic** | **Qualifier** | **PT (%)** | **Not PT (%)** | **Peason Chi-Square** | **P value** | **Holm-Bonferroni corrected threshold** | **Significance after multiple tests correction** |
| --- | --- | --- | --- | --- | --- | --- | --- |
| Shell thickness | Not reported | 4 (6.6) | 78 (8.3) | 7.300 | 0.398 | 0.003 | No |
|  | 2 mm | 2 (3.3) | 35 (3.7) |  |  |  |  |
|  | 2.5 mm | 2 (3.3) | 58 (6.2) |  |  |  |  |
|  | 3 mm | 36 (59) | 506 (53.9) |  |  |  |  |
|  | 3.5 mm | 14 (23) | 136 (14.5) |  |  |  |  |
|  | 4 mm | 2 (3.3) | 83 (8.8) |  |  |  |  |
|  | 4.5 mm | 1 (1.6) | 40 (4.3) |  |  |  |  |
|  | 5 mm | 0 (0) | 3 (0.3) |  |  |  |  |
| Shell style | Mod Root | 24 (39.3) | 590 (62.8) | 51.127 | < 0.001 | 0.002 | Yes |
|  | Sagittal Plane | 10 (16.4) | 124 (13.2) |  |  |  |  |
|  | Medial STJ | 23 (37.7) | 86 (9.2) |  |  |  |  |
|  | Other | 4 (6.6) | 139 (14.8) |  |  |  |  |
| Varus cast correction | No | 8 (13.1) | 352 (37.5) | 14.767 | < 0.001 | 0.002 | Yes |
|  | Yes | 53 (86.9) | 587 (62.5) |  |  |  |  |
| Valgus cast correction | No | 61 (100) | 883 (94) | 3.854 | 0.050 | 0.002 | No |
|  | Yes | 0 (0) | 56 (6) |  |  |  |  |
| Kirby Skive | No | 21 (34.4) | 623 (66.3) | 25.457 | < 0.001 | 0.002 | Yes |
|  | Yes | 40 (65.6) | 316 (33.7) |  |  |  |  |
| Heel raise | No | 59 (96.7) | 875 (93.2) | 1.162 | 0.281 | 0.003 | No |
|  | Yes | 2 (3.3) | 64 (6.8) |  |  |  |  |
| 1^st^ Ray cutout | No | 53 (86.9) | 771 (82.1) | 0.901 | 0.342 | 0.003 | No |
|  | Yes | 8 (13.1) | 168 (17.9) |  |  |  |  |
| Lateral side high | No | 51 (83.6) | 753 (80.2) | 0.424 | 0.515 | 0.003 | No |
|  | Yes | 10 (16.4) | 186 (19.8) |  |  |  |  |
| Medial side high | No | 49 (80.3) | 784 (83.5) | 0.413 | 0.521 | 0.003 | No |
|  | Yes | 12 (19.7) | 155 (16.5) |  |  |  |  |
| Deep heel cup | No | 37 (60.7) | 603 (64.2) | 0.315 | 0.574 | 0.004 | No |
|  | Yes | 24 (39.3) | 336 (35.8) |  |  |  |  |
| Shallow heel cup | No | 56 (91.8) | 876 (93.3) | 0.200 | 0.655 | 0.004 | No |
|  | Yes | 5 (8.2) | 63 (6.7) |  |  |  |  |
| Plantar fascia groove | No | 60 (98.4) | 877 (93.4) | 2.390 | 0.122 | 0.002 | No |
|  | Yes | 1 (1.6) | 62 (6.6) |  |  |  |  |
| Forefoot lateral wedge | No | 52 (85.2) | 730 (77.7) | 1.892 | 0.169 | 0.002 | No |
|  | Yes | 9 (14.8) | 209 (22.3) |  |  |  |  |
| Medial forefoot wedge | No | 45 (73.8) | 837 (89.1) | 12.996 | < 0.001 | 0.002 | Yes |
|  | Yes | 16 (26.2) | 102 (10.9) |  |  |  |  |
| Reverse mortons extension | No | 56 (91.8) | 854 (90.9) | 0.051 | 0.821 | 0.006 | No |
|  | Yes | 5 (8.2) | 85 (9.1) |  |  |  |  |
| Kinetic wedge | No | 60 (98.4) | 927 (98.7) | 0.058 | 0.809 | 0.006 | No |
|  | Yes | 1 (1.6) | 12 (1.3) |  |  |  |  |
| Metatarsal dome | No | 58 (95.1) | 875 (93.2) | 0.330 | 0.566 | 0.003 | No |
|  | Yes | 3 (4.9) | 64 (6.8) |  |  |  |  |
| PMP | No | 58 (95.1) | 884 (94.1) | 0.092 | 0.761 | 0.005 | No |
|  | Yes | 3 (4.9) | 55 (5.9) |  |  |  |  |
| Plantar cover | No | 58 (95.1) | 883 (94) | 0.113 | 0.737 | 0.004 | No |
|  | Yes | 3 (4.9) | 56 (6) |  |  |  |  |
| Heel Cushion | No | 59 (96.7) | 825 (87.9) | 4.387 | 0.036 | 0.002 | No |
|  | Yes | 2 (3.3) | 114 (12.1) |  |  |  |  |
| Arch pad | No | 42 (68.9) | 836 (89) | 21.773 | < 0.001 | 0.002 | Yes |
|  | Yes | 19 (31.1) | 103 (11) |  |  |  |  |
| Mortons extension | No | 60 (98.4) | 918 (97.8) | 0.095 | 0.758 | 0.005 | No |
|  | Yes | 1 (1.6) | 21 (2.2) |  |  |  |  |
| Symmetrical | No | 40 (65.6) | 385 (41) | 14.153 | < 0.001 | 0.002 | Yes |
|  | Yes | 21 (34.4) | 554 (59) |  |  |  |  |

**Ankle sprain**

| **Characteristic** | **Qualifier** | **ASpr (%)** | **Not ASpr (%)** | **Peason Chi-Square** | **P value** | **Holm-Bonferroni corrected threshold** | **Significance after multiple tests correction** |
| --- | --- | --- | --- | --- | --- | --- | --- |
| Shell thickness | Not reported | 1 (2) | 81 (8.5) | 12.273 | 0.092 | 0.002 | No |
|  | 2 mm | 2 (4) | 35 (3.7) |  |  |  |  |
|  | 2.5 mm | 0 (0) | 60 (6.3) |  |  |  |  |
|  | 3 mm | 32 (64) | 510 (53.7) |  |  |  |  |
|  | 3.5 mm | 4 (8) | 146 (15.4) |  |  |  |  |
|  | 4 mm | 7 (14) | 78 (8.2) |  |  |  |  |
|  | 4.5 mm | 4 (8) | 37 (3.9) |  |  |  |  |
|  | 5 mm | 0 (0) | 3 (0.3) |  |  |  |  |
| Shell style | Mod Root | 31 (62) | 583 (61.4) | 6.219 | 0.101 | 0.003 | No |
|  | Sagittal Plane | 5 (10) | 129 (13.6) |  |  |  |  |
|  | Medial STJ | 2 (4) | 107 (11.3) |  |  |  |  |
|  | Other | 12 (24) | 131 (13.8) |  |  |  |  |
| Varus cast correction | No | 27 (54) | 333 (35.1) | 7.401 | 0.007 | 0.002 | No |
|  | Yes | 23 (46) | 617 (64.9) |  |  |  |  |
| Valgus cast correction | No | 38 (76) | 906 (95.4) | 33.707 | < 0.001 | 0.002 | Yes |
|  | Yes | 12 (24) | 44 (4.6) |  |  |  |  |
| Kirby Skive | No | 36 (72) | 608 (64) | 1.326 | 0.250 | 0.004 | No |
|  | Yes | 14 (28) | 342 (36) |  |  |  |  |
| Heel raise | No | 48 (96) | 886 (93.3) | 0.577 | 0.447 | 0.007 | No |
|  | Yes | 2 (4) | 64 (6.7) |  |  |  |  |
| 1^st^ Ray cutout | No | 39 (78) | 785 (82.6) | 0.703 | 0.402 | 0.005 | No |
|  | Yes | 11 (22) | 165 (17.4) |  |  |  |  |
| Lateral side high | No | 20 (40) | 784 (82.5) | 54.513 | < 0.001 | 0.002 | Yes |
|  | Yes | 30 (60) | 166 (17.5) |  |  |  |  |
| Medial side high | No | 45 (90) | 788 (82.9) | 1.698 | 0.193 | 0.003 | No |
|  | Yes | 5 (10) | 162 (17.1) |  |  |  |  |
| Deep heel cup | No | 27 (54) | 613 (64.5) | 2.284 | 0.131 | 0.003 | No |
|  | Yes | 23 (46) | 337 (35.5) |  |  |  |  |
| Shallow heel cup | No | 50 (100) | 882 (92.8) | 3.840 | 0.050 | 0.003 | No |
|  | Yes | 0 (0) | 68 (7.2) |  |  |  |  |
| Plantar fascia groove | No | 49 (98) | 888 (93.5) | 1.649 | 0.199 | 0.003 | No |
|  | Yes | 1 (2) | 62 (6.5) |  |  |  |  |
| Forefoot lateral wedge | No | 26 (52) | 756 (79.6) | 21.193 | < 0.001 | 0.002 | Yes |
|  | Yes | 24 (48) | 194 (20.4) |  |  |  |  |
| Medial forefoot wedge | No | 48 (96) | 834 (87.8) | 3.077 | 0.079 | 0.002 | No |
|  | Yes | 2 (4) | 116 (12.2) |  |  |  |  |
| Reverse mortons extension | No | 43 (86) | 867 (91.3) | 1.607 | 0.205 | 0.003 | No |
|  | Yes | 7 (14) | 83 (8.7) |  |  |  |  |
| Kinetic wedge | No | 50 (100) | 937 (98.6) | 0.693 | 0.405 | 0.006 | No |
|  | Yes | 0 (0) | 13 (1.4) |  |  |  |  |
| Metatarsal dome | No | 48 (96) | 885 (93.2) | 0.614 | 0.433 | 0.006 | No |
|  | Yes | 2 (4) | 65 (6.8) |  |  |  |  |
| PMP | No | 49 (98) | 893 (94) | 1.391 | 0.238 | 0.004 | No |
|  | Yes | 1 (2) | 57 (6) |  |  |  |  |
| Plantar cover | No | 49 (98) | 892 (93.9) | 1.442 | 0.230 | 0.004 | No |
|  | Yes | 1 (2) | 58 (6.1) |  |  |  |  |
| Heel Cushion | No | 47 (94) | 837 (88.1) | 1.610 | 0.205 | 0.003 | No |
|  | Yes | 3 (6) | 113 (11.9) |  |  |  |  |
| Arch pad | No | 49 (98) | 829 (87.3) | 5.112 | 0.024 | 0.002 | No |
|  | Yes | 1 (2) | 121 (12.7) |  |  |  |  |
| Mortons extension | No | 50 (100) | 928 (97.7) | 1.184 | 0.277 | 0.005 | No |
|  | Yes | 0 (0) | 22 (2.3) |  |  |  |  |
| Symmetrical | No | 23 (46) | 402 (42.3) | 0.264 | 0.608 | 0.008 | No |
|  | Yes | 27 (54) | 548 (57.7) |  |  |  |  |
